# Reticulate sympatric speciation in Cameroonian crater lake cichlids

**DOI:** 10.1186/1742-9994-1-5

**Published:** 2004-10-26

**Authors:** Ulrich K Schliewen, Barbara Klee

**Affiliations:** 1 Department of Ichthyology, Bavarian State Collection of Zoology (ZSM), Münchhausenstr. 21, 81247 Munich, Germany

## Abstract

**Background:**

Traditionally the rapid origin of megadiverse species flocks of extremely closely related species is explained by the combinatory action of three factors: Disruptive natural selection, disruptive sexual selection and partial isolation by distance. However, recent empirical data and theoretical advances suggest that the diversity of complex species assemblages is based at least partially on the hybridization of numerous ancestral allopatric lineages that formed hybrids upon invasion of new environments. That reticulate speciation within species flocks may occur under sympatric conditions after the primary formation of species has been proposed but not been tested critically.

**Results:**

We reconstructed the phylogeny of a complex cichlid species flock confined to the tiny Cameroonian crater lake Barombi Mbo using both mitochondrial and nuclear (AFLP) data. The nuclear phylogeny confirms previous findings which suggested the monophyly and sympatric origin of the flock. However, discordant intra-flock phylogenies reconstructed from mitochondrial and nuclear data suggest strongly that secondary hybridization among lineages that primarily diverged under sympatric conditions had occurred. Using canonical phylogenetic ordination and tree-based tests we infer that hybridization of two ancient lineages resulted in the formation of a new and ecologically highly distinct species, *Pungu maclareni*.

**Conclusions:**

Our findings show that sympatric hybrid speciation is able to contribute significantly to the evolution of complex species assemblages even without the prior formation of hybrids derived from allopatrically differentiated lineages.

## Background

Recent empirical data and theoretical advances suggest that the diversity of complex species assemblages is based at least partially on the hybridization of numerous ancestral allopatric lineages that formed hybrids upon invasion of new environments [1-5]. A growing amount of studies show that cytoplasmatic (mitochondrial or chloroplast) gene phylogenies of recent diverse species radiations often conflict with phylogenies based on numerous nuclear genes [3,5-9]. Theoretical arguments as well as empirical evidence from hybrid zones predict that in newly colonized habitats the effect of transgressive segregation, i.e. the generation of extreme traits in hybrid populations, may lead to a drastically increased phenotypic variation. This effect may in turn serve as a substrate for evolution of novel adaptive traits [2,10-12]. These arguments in combination with an increasing body of evidence showing that species resulting from interspecific hybridization are common in plants [13] and highly probable in animals [5,7,9-14] gave rise to hypotheses about a prominent role of hybridization for the evolution of adaptive radiations [6]. Both, the initial formation of hybrid swarms of originally allopatric populations meeting in a newly colonized habitat ("hybrid swarm origin hypothesis") as well as secondary hybridization of *in situ *diverged lineages ("syngameon hypothesis") could possibly explain the rapid formation of megadiverse species flocks. The scenario may either involve secondary localized hybridization, i.e. hybridization of parapatric ("microallopatric") lineages within the geographical range of the primary radiation, or alternatively hybridization of sympatrically diverged lineages. Distinguishing between these two alternatives is central for the understanding of the processes that lead to the evolution of megadiversity. The first alternative predicts that increased species richness due to hybridization is dependent primarily on the spatial scale and the accompanied possibility to establish localized metapopulations. The second alternative predicts that hybridisation can aid the build-up of diversity even under fully sympatric conditions. Several studies, some published, some in preparation support the hybrid swarm origin hypothesis for some species assemblages endemic to comparatively large areas [3-5,8] but a critical evaluation of the syngameon hypotheses rests on the ability to test for sympatric hybrid speciation.

However, despite increasing evidence for sympatric speciation, uncontested examples remain rare and rarely go beyond the formation of single species-pairs [15,16]. In addition, evidence for sympatric speciation of complex species-assemblages is often based on mitochondrial phylogenies of limited taxon-sampling. This is problematic as mitochondrial phylogenies or those based on few nuclear loci may obscure true species phylogenies either due to introgressive hybridization among already established species or due to incomplete lineage sorting during rapid speciation. Hence it is not surprising, that studies applying several nuclear markers occasionally yield phylogenetic hypotheses about the origin and pattern of sympatric species assemblages which contrast with mitochondrial hypotheses. As a consequence of the uncertainty about phylogenetic relationships among members of complex species flocks questions about the processes that contribute to sympatric speciation remain difficult to test due to the lack of appropriate model systems.

Until recently the phylogeny of mitochondrial lineages of the cichlid species flock of crater lake Barombi Mbo (Cameroon) [17] was considered as one of the best examples for sympatric speciation [15]. According to this phylogeny which was based on haplotypes of single specimens, the monophyly of the 11 endemic species suggested strongly that they had formed after a single colonisation by a riverine founder species, *Sarotherodon galilaeus*. Because the lake's conical basin is only 2.15 km in diameter, because there are no migration-barriers along the shore, and because the lake is isolated from nearby river systems by cataracts of its outflow, allopatric scenarios for the origin and diversification of the flock were ruled out. However, in the light of the aforementioned methodological drawbacks of mitochondrial phylogenies, both the monophyly-hypothesis for the Barombi flock and the relationships among its 11 endemic species are worth to be reevaluated.

The ability to score thousands of amplified fragment-length polymorphisms (AFLPs) has created a powerful possibility for the phylogenetic reconstruction of rapidly originated species flocks. It has been successfully applied to a limited number of taxa belonging to the Lake Malawi and Lake Victoria haplochromine species flocks and revealed previously undetectable phylogenetic patterns including those supporting the hybrid swarm origin hypothesis [4,18,19]. In this study, we tested hypotheses about sympatric speciation with a focus on hybridization by applying a combination of mitochondrial DNA-sequencing and AFLP-genotyping as well as a set of recently proposed analytical tools [20] to the phylogenetic analysis of a complete and complex species flock.

## Results

### Mitochondrial phylogenetic inferences

**Figure 1:**
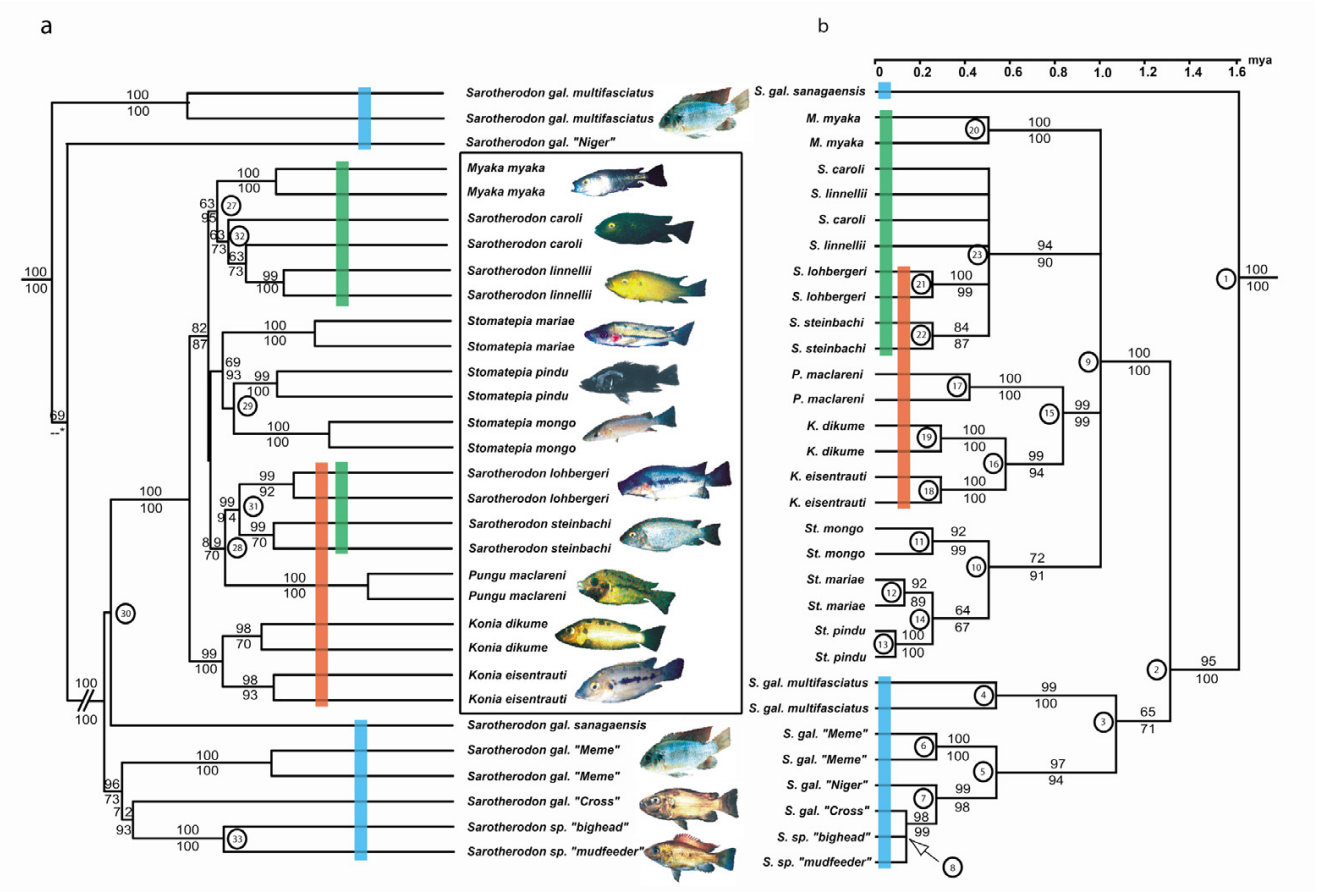
Phylogenetic tree (a) based on AFLP-data and chronogram (b) based mtDNA data of all Barombi Mbo cichlids (inside blue box), reference specimens of the closest riverine ancestor of the Barombi flock, *Sarotherodon galilaeus*, and two undescribed *Sarotherodon *from Lake Ejagham (*S. sp. "bighead" and S. sp. "mudfeeder"*). Photographs refer to the taxon to the left, different populations of *S. galilaeus *are depicted by two identical photographs only. (a) Numbers at nodes in the AFLP-tree are bootstrap-values (%) of tree reconstructions using the pruned (above) and unpruned (below) AFLP data set. Topologies were identical except for the position of *S. g. "Niger"*, which was sistergroup to *S. g. multifasciatus *when using the unpruned data (bootstrap value: 86). Long terminal branches in the original phylogram were cut to identical lengths for graphical reasons; interior branch lengths are as in the original phylogram. (b) Numbers at nodes in the mtDNA-chronogram refer to bootstrap-values of the ML (above) and MP (below) tree reconstructions. The absolute time scale above the tree is based on the maximum age of the Barombi Mbo crater formation of 1.0 mya [56]. Encircled numbers mark nodes referring to phylogenetic groups tested with PCO (see Table 1). Red, blue and green shaded boxes unite hypothetical ancient syngameons as deduced from the conflict between mtDNA- and AFLP-based phylogenetic hypotheses. Further details of tree reconstruction see *Methods*.

We obtained a DNA sequence-alignment with 2553 bp including two complete mitochondrial genes, NADH dehydrogenase subunit 2 (ND2) and cytochrome b (cytb), partial proline tRNA as well as from part of the control region from all Barombi species (two samples per species) and relevant *S. galilaeus *populations (one to two samples per population). 2191 sites of the alignment were constant, 198 variable characters were parsimony-uninformative and the number of parsimony-informative characters was 164. Empirical base frequencies in this data set were A = 0.2721; C = 0.3271; G = 0.1268; T = 0.2740. Bootstrapped Maximum Parsimony (MP), Maximum Likelihood (ML) and Neighbour Joining (NJ) trees all recovered identical 50%-majority rule consensus-trees (figure 1). As the sistergroup to the monophyletic Barombi flock a *Sarotherodon galilaeus *clade was recovered which includes all west African populations except *S. galilaeus sanagaensis *which emerged as the sistergroup to all other ingroup taxa. Within the Barombi Mbo flock four lineages were recovered with high bootstrap support, one containing the predators of genus *Stomatepia*, one combining the fine-particel feeders of the genus *Sarotherodon*, one consisting only of the dwarf zooplanctivore *Myaka *and one containing the macro-invertebrate or eggfeeding sistertaxa of the genus *Konia *plus the highly specialized spongivore *Pungu*. For taxa represented by more than one sample, all conspecific samples grouped together except those of the morphologically merely distinguishable *S. caroli *and *S. linnellii*. A rough time estimate as deduced from the ultrametric tree (chronogram) [21] derived from non parametric rate smoothening (NPRS) of bootstrapped ML-distances suggests that all four lineages almost simultaneously came into existence, which must have taken place approx. 1 myr years ago. Soon after this primary radiation, the divergence of the *Pungu *haplotypes from *Konia *took place, while all other clades radiated into several species much later. A 94 sample data set with only cytochrome b and partial proline tRNA sequences (3 to 7 samples for the Barombi taxa, and 1 to 7 for all other; 1212 bp with 1003 constant characters, 59 parsimony-uninformative and 150 parsimony-informative characters) confirmed the previous findings for the Barombi flock and suggests that lineage sorting between the four large clades is complete (for a Neighbour Joining Tree see Additional File 1). However, between species within the clades lineage sorting was only complete for a subset of taxa (*Pungu*, *Myaka*, *Konia ssp*, *S. lohbergeri *and *S. steinbachi*), but not within *Stomatepia ssp*., *S. caroli *and *S. linnellii*.

### AFLP based phylogenetic inferences

The phylogenetic reconstruction based on the same individuals and using 22 restrictive primer combinations with 3489 AFLP size fragments (3004 variable) confirmed the monophyletic origin of the Barombi Mbo flock and the monophyly of the *Stomatepia *and *Konia *clades (figure 1). However, several other phylogenetic groupings were recovered with high bootstrap support, which contrasted conspicuously with the mitochondrial phylogenetic hypothesis. *S. gal. multifasciatus *and *S. gal. "Niger" *were recovered as sistergroups to the clade containing all other ingroups including *S. gal. sanagaensis*. Within the Barombi flock *Pungu *is now sistergroup to a *Sarotherodon *subclade (*S. steinbachi *and *S. lohbergeri*), *Myaka *is sistergroup to the other *Sarotherodon *subclade consisting of the pelagic species *S. caroli *and *S. linnellii*. The *Konia *clade is resolved as the sistergroup to the rest of the flock. In contrast to the unresolved basal topology in the mitochondrial tree, the two species from Lake Ejagham are resolved as a monophylum which is the sistergroup to the geographically closest *S. galilaeus *population from the river Cross. An additional data set with 3 selective amplifications but 80 samples recovered only three supraspecific nodes with moderately high bootstrap support, the monophyly of the Barombi flock, *Konia *and the node containing the rest of the Barombi flock without *Pungu*. The latter was placed intermediate between the two well supported nodes.

### Testing for sympatric reticulate speciation

Both the Shimodaira-Hasegawa and Templeton's test confirmed significantly the difference for the alternative tree topologies for each sequence and AFLP data sets, respectively (figure 1). These discordant phylogenies suggested strongly that hybridization among previously evolved lineages had taken place and that at least one taxon of the Barombi Mbo flock, *Pungu maclareni*, is the result of speciation by hybridization. By identifying the clades which contain taxa with discordant phylogenies we hypothesized that traces of three ancient hybridization events are still detectable in the multilocus AFLP data. To test for the presence of the respective phylogenetic signal for these three hypothetical ancient syngameons in the large AFLP data set, we used the recently developed method of Canonical Phylogenetic Ordination (CPO) [20]. In addition, this method is useful for differentiating between contributions to variation in the observed AFLP character pattern that were generated by the segregation of ancestral polymorphisms inherited from a common ancestor due to incomplete lineage sorting rather than by the contribution of derived characters of hybridizing lineages. This, as the contribution to the variation that is assignable to the phylogenetic group uniting the common ancestor of the hybridizing lineages (coded as phylogenetic variables) is partialled out in the CPO separately from the contribution of the phylogenetic groups characterizing the derived lineages that may have hybridized (see also Methods section).

All phylogenetic groups detected either by the mitochondrial or the AFLP data set, or formulated according to the three hypothetical syngameons were coded as phylogenetic variables and their explanatory value for the variance in the AFLP data set was tested (table 1). As a result, almost all supraspecific clades which were not conflicting among the two data sets were recovered as contributing significantly to the variation in the data set. Conflicting clades, which were either found in the mitochondrial or AFLP phylogenetic hypothesis, yielded non-significant results except for the AFLP based nodes uniting (1) all *S. galilaeus *and lake taxa without *S. gal multifasciatus *and *S. gal "Niger" *and (2) the node of the AFLP based hypothesis of the monophyly of the Lake Ejagham sister pair. Of the three hypothetical syngameons, the one uniting *Pungu *with its potential ancestor clades represented now by the *Konia ssp *and *S. steinbachi *and *S. lohbergeri *contributed significantly to the variation in the AFLP data set, whereas the other two did not.

**Figure 2:**
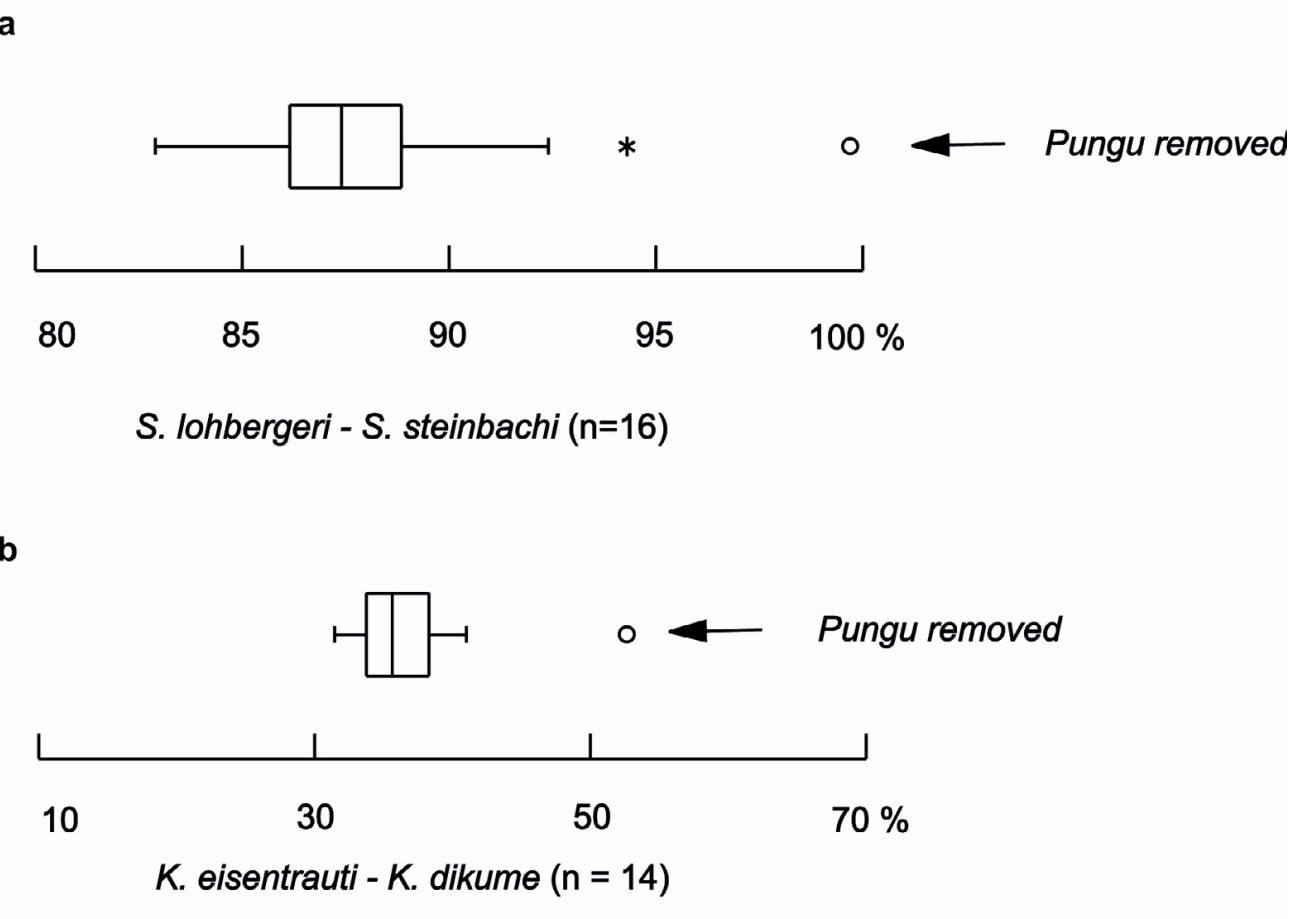
Box-plots of the distribution of %-bootstrap support values for the nodes uniting the two *Konia *species (Figs. 2a) or *Sarotherodon lohbergeri *and *S. steinbachi *(Figs. 2b) after iterative removal of single species or taxon groups. Values are based on 2000 bootstrap replicates in the AFLP-based tree reconstruction using the Link et al algorithm [48]; they are based on either the 32-sample/2355 loci dataset for the *S. lohbergeri*/*S. steinbachi *split or on the 80 sample / 530 loci dataset for the *Konia *split. Outside (*) and far outside values (°) are plotted as asterisks and circles, respectively. Arrows denote far outside values resulting in a distinctly higher bootstrap support for the two clades after exclusion of *Pungu maclareni*. n refers to the number out of 18 maximum possible removal experiments. Removal of *Pungu *did not result in outside or far outside bootstrap support values for any other node out of 30 nodes tested (Additional information concerning far outside values yielded for other nodes see Additional File 2).

As a final test for the putative hybrid origin of *Pungu*, a tree-based method as outlined in Seehausen [6] was performed in order to test for homoplasy excess introduced by this potential hybrid taxon in the AFLP-data set (figure 2). Only the removal of *Pungu *resulted in far outlying higher support values for the respective nodes, whereas all other removals resulted in much lower values (Fig. 2). In addition, the other nodes tested were not affected by the removal of *Pungu*. Interestingly, the removal of several other taxa produced far outlier values in other nodes. However, these removals concerned part of the two hypothetical syngameons that were not supported significantly in the CPO, which were therefore not explicitly tested. The removal of *Konia dikume *resulted in an increased bootstrap support for the clade uniting *Konia *and *Pungu *in the analysis of the 530 loci data set and an accompanying though weaker signal in the *S. caroli*/*S.linnellii *clade. The removal of *S. caroli *resulted in a distinct although weak increase of the bootstrap support for the *Myaka*/*S. linnellii *node, the exclusion of both *Myaka *and *Konia eisentrauti *increased the bootstrap support for the *S. linnellii/S. caroli *node. Finally, the removal of *S. mariae *increased the support for the *S. pindu/S. mongo *split strongly. Among the riverine populations of *S. galilaeus*, the removal of *S. gal. "Meme" *increased strongly the value for the clade uniting the *S. gal. "Cross" *specimen with the two endemic species from Lake Ejagham (Details in Additional File 2). These additional findings suggest that the comparative phylogenetic analysis of mtDNA and AFLP data alone was not sufficient to uncover all possible hybridization events.

## Discussion

Our results demonstrate that the sympatric origin of a diverse and complex species flock was aided substantially by reticulate evolution among lineages that emerged in a much smaller primary radiation. Our data suggest that at least one out of 11 taxa of the species flock in Lake Barombi Mbo, *Pungu maclareni*, is the result of speciation by hybridization. On the other hand, conflicts among mitochondrial and nuclear data sets as well as results of the homoplasy excess tests suggest that in the course of the evolution of the flock hybridization must have taken place among several additional Barombi taxa, too.

According to the ultrametric time-calibrated tree ("chronogram") based on the well supported phylogeny of mitochondrial haplotypes in the lake, the primary radiation in Barombi Mbo resulted into the almost instantaneous split into four distinct lineages approximately one million years ago. The accumulation of numerous apomorphic characters that support these mitochondrial lineages suggests strongly that they represented reproductively isolated species at that time. Only in an advanced stage of species flock formation and after considerable time had elapsed, their cohesion was broken partially by hybridization events between these lineages. However, according to the chronogram the ancestral mitochondrial clades that contributed, for example, to the hybrid origin of *Pungu *continued to accumulate apomorphic characters well after the origin of *Pungu*. This suggests that the species status of the ancient hybridizing lineages in terms of sufficient reproductive isolation must have allowed for their ongoing genetic cohesion and accompanied coalescence of haplotypes before additional speciation events took place.

Traditionally the rapid origin of megadiverse species flocks of extremely closely related species was explained by the combinatory action of three factors: Disruptive natural selection, disruptive sexual selection and partial isolation by distance [22-24]. Although introgression among species is known for many fish species [7,9,25-27] and although reticulate evolution and hybrid origins of species are well documented in plants [14], it is only of recent that hybridization has been proposed to play a major role in generating diversity in animals in general [27-30] and in "explosive" speciation in species flocks in particular [6]. Especially in newly colonized habitats with increased ecological opportunities, secondary hybridization of primarily diverged lineages may provide rapidly sources of heritable advantageous variation by producing additional adaptive diversity through recombination of functional genotypes. Interestingly, the species with the most likely hybrid origin in Lake Barombi Mbo, *Pungu maclareni*, represents an ecologically highly specialized ecotype. Both its peculiar dentition and the accompanying hypertrophic jaw-muscles are unique not only in Barombi Mbo but in cichlids in general [31]. Accordingly, one putative second species with a hybrid genome in the lake, *Konia dikume*, ranks among the most unusual cichlids as it is the single species which is able to exploit chironimid larvae in the almost oxygen-free deep water due to its extremely high haemoglobin concentration in its blood [32]. In the light of our findings we hypothesize that hybridization produced these extreme phenotypes by transgressive segregation which allowed the exploitation of extreme niches. This supports the notion that speciation by hybridization is not only able to produce additional random variation but may significantly increase the ecological complexity in a rapidly evolving species community by providing extraordinary genetic opportunities. If indeed transgressive segregation of hybrid genotypes plays a major role in the evolution of evolutionary novelties, members of complex species-assemblages with unusual ecological adaptations should predictably turn out to be of hybrid origin more often than species with common adaptations.

## Methods

### Taxon sampling, collection of samples and deposition of vouchers

We obtained genetic data from relevant *Sarotherodon galilaeus *populations and from all species endemic to crater lakes Barombi Mbo and Ejagham, which are related to *S. galilaeus*. *Oreochromis niloticus *and *Sarotherodon melanotheron *were used as outgroups based on published information and pilot-study data confirming their outgroup status with respect to *S. galilaeus *and the investigated species flocks.

Adult specimens from Lake Barombi Mbo were collected by UKS during field visits to the lake in 2001 and 2002 and from Lake Ejagham in 1994. Identification to species is according to Trewavas et al. [33] and was straightforward except for *Sarotherodon caroli *and *S. linnellii*. Black adult breeding males of the *S. caroli/S. linnellii*-phenotype from the deepwater were identified as *S. caroli*, whereas golden males from the shallow inshore area were identified as *S. linnellii*. Fin-clips were taken from the right pectoral fin in the field directly after collection and preserved in 96% Ethanol p.A.. Samples from additional specimens belonging to different populations of *Sarotherodon galilaeus *and outgroups were either collected by UKS in the field in Cameroon or donated by others. Vouchers are deposited in the ichthyological collection of Bavarian State Collection of Zoology (ZSM). Of few *S. galilaeus*-samples, only photographs of the specimens are available and are deposited in the ZSM, too. Informations about specimens and their species identifications, geographic origin, accession numbers and information about which specimens were sequenced and AFLP typed in different data sets are provided in Additional file 3.

### Molecular Methods

#### DNA Preparation

DNA samples were isolated from approx 10 mm^2 ^fin tissue with the DNeasy™ Tissue Kit (Qiagen). DNA-quality was visually inspected under UV-light on a 0.8% agarose-gel stained with ethidium bromide. For subsequent AFLP-analysis only samples with a clearly visible high-molecular band were used. DNA-concentration was determined using the VersaFluor-Fluorometer-System (BioRad) using the stain Picogreen^® ^dsDNA Quantitation Kit (Molecular Probes). All samples were adjusted to 60 ng/μl.

#### Mitochondrial DNA Amplification and Sequencing

Two different data sets were assembled, one long data set with approx. 2550 bp including the complete NADH dehydrogenase subunit 2 (ND2), the complete cytochrome b (cytb) gene and part of the proline tRNA, and finally, one part of the control region. For this long data set only two samples per species were used, but a short data set with more individuals but only the cytochrome b and the partial proline tRNA genes was generated, too. ND2 was PCR-amplified using the primers "ND2Met" 5'-CATACCCCAAACATGTTGGT-3'"ND2Trp" 5'-GTSGSTTTTCACTCCCGCTTA-3'; a second fragment containing the cytb, proline and threonine tRNAs and the 5'-end of the control region was amplified with the primers "L14725" 5'-TGACTTGAAAAACCATCGTTG and "H16498" 5'-CCTGAAGTAGGAACCAGATG [34] ; internal sequencing primers were the newly designed "cytL640" 5'-CACGAAACCGGATCAAAC-3' for cytochrome b and "L71" 5'-TACCCCTAGCTCCCAAAGCT-3'^7 ^for the 5'-end of the control region. PCR was performed by using a PTC 220 DYAD thermocycler (MJ Research) in a 25 μl reaction volume using the Expand PCR system (Roche Diagnostics) with 25 pmol of each primer, 20 pmol of dNTPs, 12.5 pmol MgCl_2 _and 0.88 units of Taq polymerase. PCR parameters were 94°C for 4 min, 35 cycles with 94°C for 1.5 min, 55°C for 1 min, 72°C for 1.5 min, followed by a final elongation at 72°C for 3 min. PCR products were cleaned by using MinElute PCR purification kit (QIAGEN) and their DNA-concentration adjusted to 100 ng/μl. PCR-Products were then used as templates for cycle-sequencing reaction using the "Ready Reaction DyeDeoxy Terminator Cycle Sequencing Kit" (Applied Biosystems) with each of the PCR primers or internal primers. Cycle parameters were the following: 94°C, 2 min; 25 cycles of 94°C, 20 s; 52°C, 10 s; 60°C, 4 min. The sequenced product was filtered through Sephadex-G50 fine (Fluka) packed spin columns (Amersham) to remove unincorporated dye terminators, primers, and salts, and finally dried in a speed-vac. These products were resuspended, electrophoresed and analysed with an ABI PRISM™ 377XL-96 automated sequencer using a 4.25 % polyacrylamid gel (BioRad). Electrophoretic information was transcribed to sequence data using the program Genescan (PE Applied Biosystems).

Individual sequence files were edited and contigs assembled using Sequence Navigator™ (PE Applied Biosystems). Homologous protein-coding regions (ND2, cytb) were aligned manually and confirmed by translating DNA data into amino acid sequences in BioEdit [35]. The short fragment of the control-region was first aligned with default settings in Clustal W as implemented in Sequence Navigator™. No indels larger than 1 basepair (bp) were detected and alignment therefore was straightforward. All sequences were tested for an anti-G bias characteristic of the mitochondrial DNA to confirm that we have collected genuine mitochondrial DNA data [36]. Sequence data have been deposited in GenBank (for accession numbers se Additional File 3).

#### Amplified Fragment-Length Polymorphisms (AFLPs)

We followed the original protocol of the AFLP-method [37] using the AFLP™ Plant Mapping Kit (Applied Biosystems) with slight modifications of the accompanied protocol: Restriction and ligation were carried out in a single step under standardized conditions in a thermocycler (2 h at 37°C and 8 h at 16°C). 1,5 μl of the preselective amplification product were used in only 10 μl total reaction volume of the selective amplifications. The restriction enzymes used were EcoRI and MseI. Primer sequences for preselective PCR were GACTGCGTACCAATTCA and GATGAGTCCTGAGTAAC. An additional two bases were added to the 3' end for selective PCR. Analogous to the mitochondrial data set we assembled two datasets. For the long one in total 22 primer pairs were used in the following combinations and fluorescent dye-labelling (MseI-primer/ EcoRI^DYE^): TC-CA^FAM^, AT-CA^FAM^, AC-CC^NED^, TA-CA^FAM^, AA-CT^FAM^; AA-GG^JOE^; AC-CA^FAM^; AA-CA^FAM^; AG-CA^FAM^; AA-CG^JOE^; AC-CT^FAM^; AG-CT^FAM^; AT-CT^FAM^; AG-GG^JOE^; TC-CT^FAM^; TA-GG^JOE^; AG-AC^NED^; AT-AC^NED^; AG-CC^NED^; AC-AC^NED^; AT-CC^NED^; TA-CG^NED^. For the short data only the five primer pairs TC-CA^FAM^, TA-GG^JOE^, AC-CA^FAM^, AC-CC^NED^, AT-CA^FAM ^were used but typed for 80 individuals (see Additional File 3).

Selectively amplified fragments were separated on 6% LongRanger polyacrylamid gels (FMC BioProducts) with an ABI PRISM™ 377XL-96 sequencer. Fluorescent signals were detected using the GENESCAN software (Applied Biosystems) with internal size standard (GS-500 ROX; Applied Biosystems). The fluorescent threshold was set to 50 units and the correct identification of ROX-marker bands by GENESCAN was checked for all electropherograms.

Bands between 100.5 bp and 499.5 bp were scored in a first step for presence or absence using the software Binthere (developed by N. Garnhart and available through the T. Kocher laboratory . The program generates aligned spreadsheets from GENESCAN-sized AFLP-data by assigning each sized fragment to a size-class of user-defined distance to the next size-class. Using a spreadsheet routine, fragments were inferred in a second step to be homologous if they differed by no more than 1.00 bp, and if the scoring procedure identified the same size-classes whether scored from small to large size-fragments (forward) or vice versa (backward). Size-classes with inconsistent allocation of fragments according to forward and backward scoring were excluded, as well as adjacent size-classes differing by less than 0.35 bp, which corresponds to the double standard deviation of 0.15 bp of the sequencer [38]. As a result of this procedure a final 0/1 data-matrix for all scored individuals was prepared. All samples of the same primer combination were run on same gel for the 33 specimen dataset and on two gels for the 80 taxon dataset. Single unsuccessful amplifications were repeated and fitted to the data matrix using the size-class assignment criteria as outlined above.

### Phylogenetic Analyses

#### Mitochondrial Sequence Data

MrModeltest 1.1b [39], a simplified version of David Posada's "Modeltest 3.06" [40] was used to perform hierarchical likelihood ratio tests (HLRT) and to calculate approximate Akaike Information criteria (AIC) to determine the optimal nucleotide substitution models for the dataset. If the two tests did not select the same model, we chose AIC over HLRT, as AIC is a useful measure that rewards models for good fit but imposes a penalty for unnecessary parameters [41,42], which may cause erroneous phylogenetic conclusions especially in Bayesean phylogenetic analyses [43]. For the 33 sequence dataset the HLRT selected the HK+I+Γ model (α = 0.2781; proportion of invariable sites = 0) a transition/transversion (Ti/tv) ratio of 8.5662 was calculated. The AIC selected the HKY+I+ Γ model (α = 0.9468; proportion of invariable sites = 0.4264) with a transition/transversion ratio of 8.6616. For the 94 sequence dataset, the HLRT selected the GTR+ Γ model (α = 0.4014; proportion of invariable sites = 0), whereas AIC selected the GTR+I model (proportion of invariable sites = 0.5817). Empirical base frequencies in the data 2553/1212 data sets were A = 0.2443/0.2440; C = 0.3264/0.3261; G = 0.1443/0.1446; T = 0.2581/0.2852.

The AIC settings were subsequently used for Maximum Likelihood (ML) analyses and to estimate ML distances for minimum evolution (ME) analyses in the program PAUP* 4.0b1.0 (PPC/Altivec) [44] and for the 94 sequence dataset in Treefinder [45]. Maximum Parsimony (MP) analyses were conducted with heuristic searches (TBR branch swapping and MULTREES option effective; 10 random stepwise additions of taxa for 33 sequence set and simple addition for the 94 sequence set; gaps in the control region treated as a 5^th ^character). Non-parametric bootstrapping with 1000 (ME or MP analyses) or 100 (ML) pseudoreplicates was used for testing the robustness of the inferred trees. Tree topologies using the HLRT settings under ME were not different from topologies gained with AIC settings (data not shown).

A LRT [46] as implemented in PAUP* was performed with the respective 33 sequence ME tree under the AIC model assumptions with (-ln L = 6770.47061) and without (-ln L = 7120.92833) molecular clock enforced. Overall constancy of rates of evolution was rejected (chi^2 ^= 701.0154, df = 32, p = 0.001). To date cladogenetic events in the absence of rate constancy, the nonparametric rate smoothing (NPRS) method [21] as implemented in Treefinder [45] was used to construct an ultrametric tree ("chronogram") using the bootstrapped 33 sequence ML derived tree-topology and associated bootstrapped branch lengths as input.

#### AFLP Data

We used PAUP* [44] to calculate the skewness parameter g1 [47] to test for adequacy of phylogenetic signal in the 0/1-data set. g1 calculated from 1000000 random trees revealed significant non-random structure under the parsimony optimality criterion in the 22 restrictive amplifications for the complete data set: g1 -0.805, 33 samples, 3004 variable sites out of 3489 scored); g1 values were lower in the 3 restrictive amplifications data set used for some homoplasy excess tests (see below): g1 -0.298, 80 samples, 717 variable sites out of 859 scored. 2355 and 530 loci respectively were parsimony informative within *Sarotherodon galilaeus sensu lato *(excluding *Oreochromis niloticus *and *Sarotherodon melanotheron*). "Pruned" data matrices using only those parsimony informative sites were constructed for Principal Canonical Ordination and homoplasy excess tests (see below) in order to account for noise in the data potentially introduced by the distant outgroup. Pairwise genetic distances were calculated from the binary data-matrix with two different algorithms: One developed by Link et al. [48] as implemented in TREECON v.1.3b [49], which is based on shared and unique characters and ignores shared absence. This algorithm is adequate for AFLP data, since noise in the data may often be created by weak signal intensities and hence absence of band-detection despite a possible weak presence of signal. Alternatively, we calculated pair-wise distance matrices with the restriction-site program RESTDIST within the PHYLIP 3.6A2 package [50]. Trees were constructed from the Link et al.-distances with the neighbour joining (NJ) algorithm as implemented within TREECON or from the RESTDIST-distances using the Fitch-and-Margoliash-algorithm [51] with unconstrained branch length as implemented in the program FITCH within the PHYLIP 3.6A2 package [50]. Non-parametric bootstrapping was performed with 100 bootstrapped data sets analyzed 10 times with random input orders, and with local and global optimization.

#### Hypothesis testing

Alternative phylogenetic hypotheses produced as described by tree topologies based on mtDNA and AFLP data were compared with each other and statistically evaluated using either the Shimodaira-Hasegawa LRT [52] for mitochondrial data or the Templeton's Wilcoxon signed-rank test [53] for AFLP-data (both as implemented in PAUP*).

In order to test for the presence of a phylogenetic signal that possibly reflects reticulate events in the AFLP-data, two methods were applied.

First, a canonical correspondence analysis (CCA) was performed using CANOCO 4.0 [54]. This method has previously been used successfully for testing the effect of tree-like hydrogeographic data and supplementary ecological data on microsatellite allele-frequencies in freshwater fishes [55], as well as an alternative to traditional phylogenetic comparative methods [20]. A presence/absence matrix with the 2355 AFLP-characters which were parsimony-informative within the *Sarotherodon galilaeus*-clade (*S. galilaeus *sensu lato and lake endemics) provided the data-matrix to be tested. Phylogenetic hypotheses derived from mtDNA- and AFLP-analyses as well as hypothetical syngameons as derived from the conflict between the two data-sets were translated into a phylogenetic matrix by assigning binary indicator variables, each coding for the membership of investigated samples to phylogenetic groups (e.g. nodes in phylogenetic trees or hypothetical syngameons) [20,54]. 9999 full model Monte Carlo (MC) permutations were used to test whether a given phylogenetic group as coded by the indicator variables and identified by automatic forward selection of variables was significantly related to the AFLP-data pattern.

Second, a tree-based method as outlined in Seehausen [6] was performed in order to test for homoplasy excess introduced by potential hybrid taxa in the AFLP-data as suggested by the mtDNA-AFLP-phylogeny conflict and the CCA. Theoretically, hybrid taxa are overall intermediate to the parental taxa because they carry a mosaic of parental characters. Consequently, the inclusion of a hybrid taxon into a multilocus based phylogeny estimate introduces an excess of homoplasies and therefore conflict in the subset of clades that contributed to hybridization. Removal of the putative hybrid taxon should therefore decrease the amount of homoplasies and hence increase support for those nodes that unite descendants from taxa which gave rise to a hybrid taxon. In contrast, removal of a non-hybrid taxon should not affect support for the respective nodes. We computed Link et al bootstrap-supports (2000 replicates) for the nodes uniting *Sarotherodon steinbachi *and *S. lohbergeri *in the 2355 loci data with n = 16 experiments (each taxon removed once). Analogous support values for the node uniting the *Konia eisentrauti *and *K. dikume *were computed with the 530 loci data set with n = 14 experiments, because bootstrap support in the larger data set was always larger than 98.85% and identical runs yielded values differing by more than 1.15 % (data not shown). By reducing the number of loci but increasing the number of samples we obtained a meaningful distribution of bootstrap support values for that node.

## Authors' contributions

UKS designed the study, carried out the field work, part of the lab work, the major part of the data analysis and wrote the manuscript. BK carried out the major part of the lab work and carried out parts of the data analysis. Both authors read and approved the final manuscript.

**Table 1:** Results of canonical phylogenetic ordination of AFLP data

		Variation explained¶		
	Node ¥	Marginal effects† λ_1_	Conditional effects‡ λ_a_	P-Value#

**Phylogenetic groups according to mtDNA-based phylogeny**				
S. galilaeus sensu lato incl. Barombi taxa *	1	0.15	0.10	0.000
*S. galilaeus sensu lato *w/o *S. g. sanagaensis incl. Barombi taxa*	2	0.12	0.05	n.s.
*S. galilaeus w/o S. g. sanagaensis excluding Barombi taxa*	3	0.15	-	n.s.
S. g. multifasciatus *	4	0.18	0.13	0.000
*S. galilaeus *w/o *S. g. sanagaensis *and *S. g. multifasciatus*	5	0.12	-	n.s.
*S. galilaeus *"Meme" *	6	0.10	0.08	0.000
"Cross-clade" + *S. g. *"*Niger*"	7	0.10	-	n.s.
"Cross-clade" *	8	0.09	0.07	0.001
"Barombi Mbo clade" *	9	0.18	0.11	0.000
Stomatepia ssp.*	10	0.08	0.06	0.000
St. mongo *	11	0.07	0.05	0.054
St. mariae *	12	0.06	0.05	0.008
St. pindu *	13	0.05	-	n.s.
*St. mariae *– *St. pindu*	14	0.06	-	n.s.
*Pungu maclareni *– *Konia ssp.*	15	0.07	0.05	0.025
Konia ssp. *	16	0.06	0.06	0.001
Pungu maclareni *	17	0.06	-	n.s.
Konia eisentrauti *	18	0.05	0.04	n.s.
Konia dikume *	19	0.05	-	n.s.
Myaka myaka *	20	0.05	0.04	n.s.
Sarotherodon lohbergeri *	21	0.06	0.04	n.s.
Sarotherodon steinbachi *	22	0.05	-	n.s.
"Barombi *Sarotherodon *clade"	23	0.08	-	n.s.
**Phylogenetic groups according to AFLP-phylogeny**				
*Myaka *+ *S. caroli*/*S.linnellii*	27	0.07	-	n.s.
*Pungu *+ *S. lohbergeri*/*S. steinbachi*	28	0.07	-	n.s.
*St. mongo *+ *St. pindu*	29	0.07	-	n.s.
*S. galilaeus s. l. incl. Barombi taxa w/o S.g.multifasciatus *and *S.g. "Niger"*	30	0.21	0.21	0.000
*S. lohbergeri *+ *S. steinbachi*	31	0.06	-	n.s.
*S. linnellii *+ *S. caroli*	32	0.06	-	n.s.
*S. sp. "mudfeeder" *+ *S. sp. "bighead"*	33	0.08	0.05	0.013
**Hypothetical ancient syngameons according to conflict between mtDNA-based and AFLP-based phylogenetic hypotheses**	groups ¥¥			
*P. maclareni *+ *Konia ssp. *+ *S. lohbergeri *+ *S. steinbachi*	red (24)	0.08	0.06	0.002
*M. myaka *+ *S. caroli *+ *S. linnellii *+ *S. lohbergeri *+ *S. steinbachi*	green (25)	0.09	-	n.s.
*S. g. sanagaensis *+ *S. galilaeus *w/o *S. g. multifasciatus. S. g. "Niger"*	blue (26)	0.15	-	n.s.

Each phylogenetic group as identified by nodes in phylogenetic analyses of mtDNA-, or AFLP-data or by the conflict between the data sets (hypothetical syngameons) was tested for their explanatory value in explaining variance in the AFLP data set.

¥ Node numbers correspond to encircled node numbers for mtDNA-clades and AFLP-clades supported in Fig. 1.

¥¥ Groups refer to hypothetical syngameons as indicated by coloured blocks in Fig. 1a and b.

¶ "Variation explained" refers to eigenvalues of each phylogenetic group that explain part of the variation in the pruned AFLP-data (2355 loci / 33 samples identical to those in Fig. 1a. Sum of all unconstrained eigenvalues was 1.667, sum of all canonical eigenvalues 1.179.

† Marginal effects refer to eigenvalues ("fit") of each phylogenetic group if taken singly as the only variable on the pruned AFLP data set.

‡ Conditional effects refer to the increase in eigenvalues ("additional fit") of each phylogenetic group as selected by automatic forward selection.

# P-values refer to the significance level of the conditional effects as obtained with a Monte Carlo permutation test under the full model with 9999 random permutations.

* mtDNA-clades as indicated in Fig. 1b that are compatible with clades as identified by the AFLP phylogenetic tree in Fig. 1a.

## Supplementary Material

Additional File 1 Neighbour Joining phylogram of 94 specimens Neighbour. Joining phylogram calculated from complete sequences of the mitochondrial cytochrome b gene (1141 bp) and the partial proline tRNA gene (71 bp) sequences for 94 specimens based on the GTR+ Γ model and rooted with *Sarotherodon melanotheron *(not shown) Click here for file

Additional File 2 Additional box-plots of bootstrap supports in taxon removal experiments. Box-plots of the distribution of %-bootstrap support values for nodes that yielded additional higher outside or far outside values in taxon removal experiments (methodology compare with legend of Fig. 2 of the main text). **a. **The removal of *Konia dikume *resulted in an increased bootstrap support for the clade uniting *Konia *and *Pungu *in the analysis of the 530 loci data; set. **b. **The removal of *S. caroli *resulted in increased outside values for the bootstrap support of the **b. **the *Myaka*/*S. linnellii *node and the **c. **the clade that unites all Barombi taxa with the exclusion of *Konia*; **d. **the exclusion of both *Myaka *and *K. eisentrauti *increased the bootstrap support for the *S. linnellii/S. caroli *node; **e. **Finally, the removal of *S. mariae *increased the support for the *S. pindu/S. mongo *split strongly. **f. **Among the riverine populations of *S. galilaeus*, the removal of *S. gal. "Meme" *increased strongly the value for the clade uniting the *S. gal. "Cross" *specimen with the two endemic species from Lake Ejagham ("Cross clade"); in addition, the exclusion of *S. lohbergeri *increased the bootstrap value for the same node, albeit weakly. Click here for file

Additional File 3 Specimens genotyped, Genbank Accession Numbers and vouchers. Specimens included in the study with information on DNA-reference-numbers, voucher deposition in the Zoological State Collection Munich (ZSM), Genbank-Accession numbers and representation of specimens in two AFLP-data sets. Click here for file

## References

[B1] Rieseberg LH, Archer MA, Wayne RK (1999). Transgressive segregation, adaptation, and speciation.. Heredity.

[B2] Gilbert LE, Boggs CL, Ward BW, Ehrlich PR (2003). Adaptive novelty through introgression in *Heliconius *wing patterns: evidence for shared genetic "tool box" from synthetic hybrid zones and a theory of diversification.. Ecology and Evolution of taking Flight: Butterflies as a Model System.

[B3] Barrier M, Baldwin B, Robichaux RH, Purugganan MD (1999). Interspecific hybrid ancestry of a plant adaptive radiation: allopolyploidy of the Hawaiian silversword alliance inferred from duplicated floral homeotic genes.. Mol Biol Evol.

[B4] Seehausen O, Koetsier E, Schneider MV, Chapman LJ, Chapman CA, Knight ME, Turner GF, van Alphen JJM, Bills R (2003). Nuclear markers reveal unexpected genetic variation and a Congolese/Nilotic origin of the Lake Victoria cichlid species flock.. Proc R Soc Lond B Biol Sci.

[B5] Shaw KL (2002). Conflict between mitochondrial and nuclear DNA phylogenies of a recent species radiation: what mitochondrial reveals and conceals about modes of speciation in Hawaiian crickets.. Proc Natl Acad Sci USA.

[B6] Seehausen O (2004). Hybridisation and adaptive radiation.. Trends Ecol Evol.

[B7] Dowling TE, DeMarais BD (1993). Evolutionary significance of introgressive hybridization in cyprinid fishes.. Nature.

[B8] Beltrán M, Jiggins CD, Bull V, Linares M, McMillan WO, Mallet J, Bermingham E (2002). Phylogenetic discordance at the species boundary: gene genealogies in *Heliconius *butterflies.. Mol Biol Evol.

[B9] Salzburger W, Baric S, Sturmbauer C (2002). Speciation via introgressive hybridization in East African cichlids?. Mol Ecol.

[B10] Templeton AR (1981). Mechanisms of speciation – a population genetic approach.. Ann Rev Ecol Syst.

[B11] Burke JM, Arnold ML (2001). Genetics and the fitness of hybrids.. Ann Rev Genet.

[B12] Barton NH (2001). The role of hybridization in evolution.. Mol Ecol.

[B13] Arnold ML (1997). Natural Hybridisation and Evolution.

[B14] Smith PF, Konings A, Kornfield I (2003). Hybrid origin of a cichlid population in Lake Malawi: Implications for genetic variation and species diversity.. Mol Ecol.

[B15] Via S (2001). Sympatric speciation in animals: the ugly duckling grows up.. Trends Ecol Evol.

[B16] Schliewen UK, Rassmann K, Markmann M, Markert J, Kocher TD, Tautz D (2001). Genetic and ecological divergence of a monophyletic cichlid species pair under fully sympatric conditions in Lake Ejagham, Cameroon.. Mol Ecol.

[B17] Schliewen UK, Tautz D, Pääbo S (1994). Sympatric speciation suggested by monophyly of crater lake cichlids.. Nature.

[B18] Albertson RC, Markert JA, Danley PD, Kocher TD (1999). Phylogeny of a rapidly evolving clade: the cichlid fishes of Lake Malawi, East Africa.. Proc Natl Acad Sci USA.

[B19] Allender CJ, Seehausen O, Knight ME, Turner GF, Maclean N (2003). Divergent selection during speciation of Lake Malawi cichlid fish inferred from parallel radiations in nuptial coloration.. Proc Natl Acad Sci USA.

[B20] Giannini NP (2003). Canonical phylogenetic ordination.. Syst Biol.

[B21] Sanderson MJ (1997). A nonparametric approach to estimating divergence times in the absence of rate constancy.. Mol Biol Evol.

[B22] Sturmbauer C (1998). Explosive speciation in cichlid fishes of the African Great Lakes: a dynamic model of adaptive radiation.. J Fish Biol.

[B23] Danley PD, Kocher TD (2001). Speciation in rapidly diverging systems: lessons from Lake Malawi.. Mol Ecol.

[B24] Kocher TD (2004). Adaptive evolution and explosive speciation: the cichlid fish model.. Nature Reviews Genetics.

[B25] Wilson CC, Bernatchez L (1998). The ghost of hybrids past: fixation of arctic charr (*Salvelinus alpinus*) mitochondrial DNA in an introgressed population of lake trout (*S. namaycush*).. Mol Ecol.

[B26] Rognon X, Guyomard R (2003). Large extent of mitochondrial DNA transfer from *Oreochromis aureus *to *O. niloticus *in West Africa.. Mol Ecol.

[B27] Scribner KT, Page K, Bartron M (2001). Life history and behavioral ecology impact rates and direction of evolutionary change in fish hybrid zones: a cytonuclear perspective.. Rev Fish Biol Fisheries.

[B28] Turner GF (2002). Parallell speciation, despeciation and respeciation: implications for species definition.. Fish Fisheries.

[B29] Dowling TE, Secor CL (1997). The role of hybridization in the evolutionary diversification of animals. Ann Rev Ecol Syst.

[B30] Grant PR, Grant BR (1992). Hybridization of bird species.. Science.

[B31] Dominey WJ (1987). Sponge-eating by *Pungu maclareni*, an endemic cichlid fish from Lake Barombi Mbo, Cameroon.. Nat Geogr Res.

[B32] Green J, Corbet SA, Betney E (1973). Ecological studies in crater lakes in West Cameroon. The blood of the endemic cichlids in Barombi Mbo in relation to stratification and their feeding habits.. J Zool (Lond).

[B33] Trewavas E, Green J, Corbet SA (1972). Ecological studies on crater lakes in West Cameroon. Fishes of Barombi Mbo.. J Zool (Lond).

[B34] Kocher TD, Thomas WK, Meyer A, Edwards SV, Pääbo S, Villablanca FX, Wilson AC (1989). Dynamics of mitochondrial DNA evolution in animals: amplification and sequencing with conserved primers.. Proc Natl Acad Sci USA.

[B35] Hall TA (1999). BioEdit: a user-friendly biological sequence alignment editor and analysis program for Windows 95/98/NT.. Nucl Acids Symp Ser.

[B36] Zhang D-X, Hewitt GM (1996). Highly conserved nuclear copies of the mitochondrial control region in the desert locust *Schistocerca gregaria*: some implications for population studies.. Mol Ecol.

[B37] Vos P, Hogers R, Bleeker M, Reijans M, van de Lee T, Homes M, Frijters A, Pot J, Peleman J, Kuiper M, Zabeau M (1995). AFLP: a new technique for DNA fingerprinting.. Nucl Acids Res.

[B38] Lazaruk K, Walsh PS, Oaks F, Gilbert D, Rosenblum BB, Menchen S, Scheibler D, Wenz HM, Holt C, Wallin J (1998). Genotyping of forensic short tandem repeat (STR) systems based on sizing precision in a capillary electrophoresis instrument.. Electrophoresis.

[B39] Nylander JA (2002). MrModeltest v10b.

[B40] Posada D, Crandall KA (2001). Modeltest vers 306.

[B41] Hasegawa M (1990). Phylogeny and molecular evolution in primates.. Jpn J Genet.

[B42] Posada D, Crandall KA (1998). Modeltest: testing the model of DNA substitution.. Bioinformatics.

[B43] Erixon P, Svennblad B, Britton T, Oxelman B (2003). Reliability of Baysean posterior probabilities and bootstrap frequencies in phylogenetics.. Syst Biol.

[B44] Swofford D (2000). PAUP*. Phylogenetic analysis using parsimony (*and other methods).

[B45] Jobb G (2003). Treefinder vers Dec.

[B46] Huelsenbeck JP, Crandall KA (1997). Phylogeny estimation and hypothesis testing using maximum likelihood.. Ann Rev Ecol Syst.

[B47] Hillis DM, Huelsenbeck JP (1992). Signal, noise, and reliability in molecular phylogenetic analyses.. J Hered.

[B48] Link W, Dixkens C, Singh M, Schwall A, Melhiger AE (1995). Genetic diversity in European and Mediterranean faba bean germplasm revealed by RAPD markers.. Theor Appl Genet.

[B49] Van de Peer Y, de Wachter R (1994). TREECON for Windows: a software package for the construction and drawing of evolutionary trees for the Microsoft Windows environment.. Comp Appl Biosc.

[B50] Felsenstein WM (2001). Phylip 362 alpha.

[B51] Fitch WM, Margoliash E (1967). Construction of phylogenetic trees.. Science.

[B52] Shimodaira H, Hasegawa M (1999). Multiple comparisons of log-likelihoods with applications to phylogenetic inference.. Mol Biol Evol.

[B53] Templeton AR (1983). Phylogenetic inference from restriction endonuclease cleavage site maps with particular reference to the evolution of humans and the apes.. Evolution Int J Org.

[B54] ter Braak CJF, Smilauer P (1998). CANOCO Reference Manual and User's Guide to Canoco for Windows: Software for Canonical Community Ordination (version 4).

[B55] Angers B, Magnan P, Plante M, Bernatchez L (1999). Canonical correspondence analysis for estimating spatial and environmental effects on microsatellite gene diversity in brook charr (*Salvelinus fontinalis*).. Mol Ecol.

[B56] Cornen G, Bandet Y, Giresse P, Maley J (1992). The nature and chronostratigraphy of Quaternary pyroplastic accumulations from Lake Barombi Mbo (West-Cameroon).. J Volc Geotherm Res.

