# Supplementary figures and images for: Reticulate sympatric speciation in Cameroonian crater lake cichlids
Source: Front Zool. 2004 Oct 26;1:5. doi: 10.1186/1742-9994-1-5 (PMC544937; doi:10.1186/1742-9994-1-5)

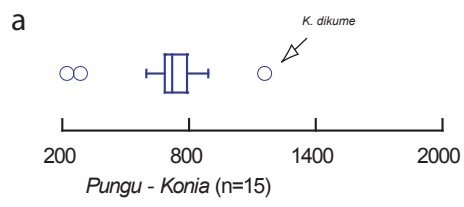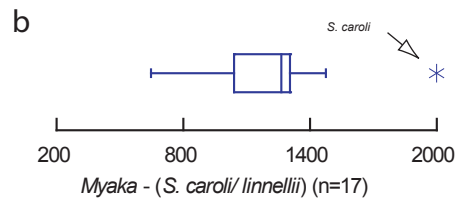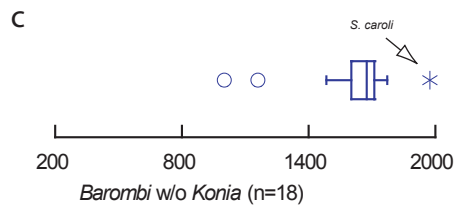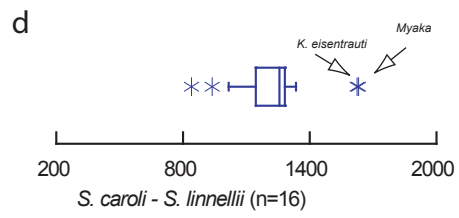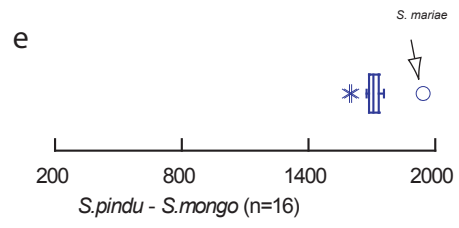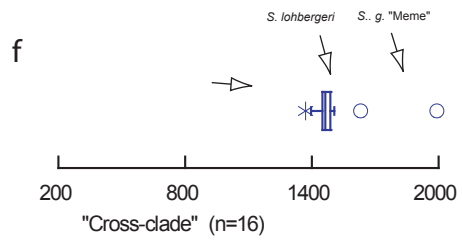

Supplement: Additional File 1 — Neighbour Joining phylogram of 94 specimens Neighbour. Joining phylogram calculated from complete sequences of the mitochondrial cytochrome b gene (1141 bp) and the partial proline tRNA gene (71 bp) sequences for 94 specimens based on the GTR+ Γ model and rooted with Sarotherodon melanotheron (not shown) [file 1742-9994-1-5-S1.pdf]

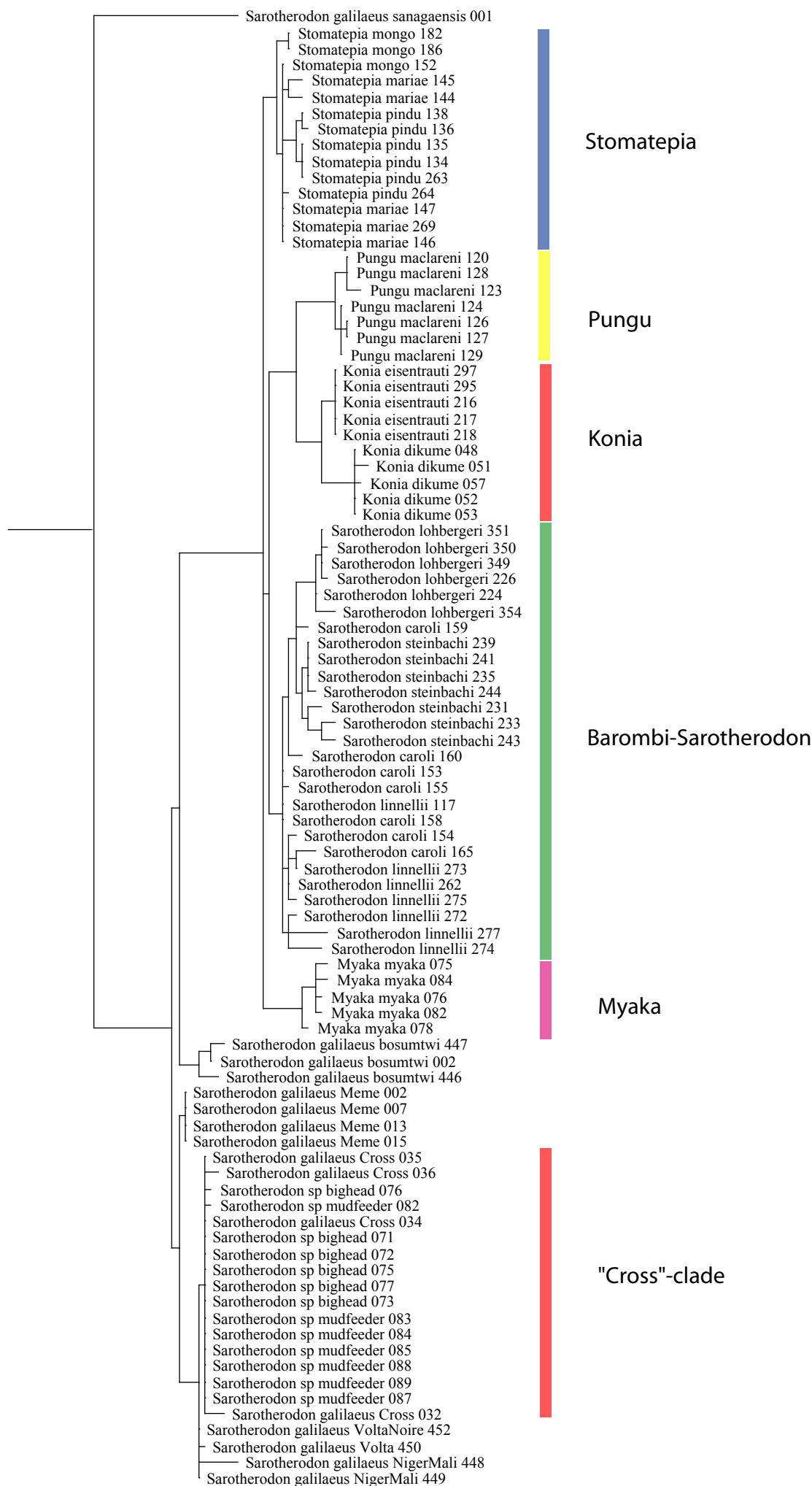

Supplement: Additional File 2 — Additional box-plots of bootstrap supports in taxon removal experiments. Box-plots of the distribution of %-bootstrap support values for nodes that yielded additional higher outside or far outside values in taxon removal experiments (methodology compare with legend of Fig. 2 of the main text). a. The removal of Konia dikume resulted in an increased bootstrap support for the clade uniting Konia and Pungu in the analysis of the 530 loci data; set. b. The removal of S. caroli resulted in increased outside values for the bootstrap support of the b. the Myaka/S. linnellii node and the c. the clade that unites all Barombi taxa with the exclusion of Konia; d. the exclusion of both Myaka and K. eisentrauti increased the bootstrap support for the S. linnellii/S. caroli node; e. Finally, the removal of S. mariae increased the support for the S. pindu/S. mongo split strongly. f. Among the riverine populations of S. galilaeus, the removal of S. gal. "Meme" increased strongly the value for the clade uniting the S. gal. "Cross" specimen with the two endemic species from Lake Ejagham ("Cross clade"); in addition, the exclusion of S. lohbergeri increased the bootstrap value for the same node, albeit weakly. [file 1742-9994-1-5-S2.pdf]
