# Supplementary material for: Reticulate sympatric speciation in Cameroonian crater lake cichlids
Source: Front Zool. 2004 Oct 26;1:5. doi: 10.1186/1742-9994-1-5 (PMC544937; doi:10.1186/1742-9994-1-5)
Supplement: Additional File 3 — Specimens genotyped, Genbank Accession Numbers and vouchers. Specimens included in the study with information on DNA-reference-numbers, voucher deposition in the Zoological State Collection Munich (ZSM), Genbank-Accession numbers and representation of specimens in two AFLP-data sets. [file 1742-9994-1-5-S3.doc]

| Specimens included with information on DNA-reference-numbers, voucher deposition in the Zoologische Staatssammlung München (ZSM), Genbank-Accession numbers and representation of specimens in two AFLP-data sets. | | | | | | | | |
| --- | --- | --- | --- | --- | --- | --- | --- | --- |
|  |  |  |  | Mitochondrial sequences (Genbank Acc.-No.) | | | AFLP amplifications | |
| ***Species*** | **Specimen**  **reference No** | **ZSM Lot-No** | **Location** | **Cytb (and partial Proline tRNA)** | **ND2** | **D-Loop** | **22 amplifications** | **5 amplifications** |
| *Stomatepia mongo* | CAM01_182 | ZSM 29837-38 | Lake Barombi Mbo, Cameroon | AJ844919 | AJ845094 | AJ845140 | X | X |
| *Stomatepia mongo* | CAM01_186 |  |  | AJ844920 | AJ845095 | AJ845141 | X | X |
| *Stomatepia mongo* | CAM01_152 |  |  | AJ844921 | - | - | - | X |
| *Stomatepia mongo* | CAM01_110 |  |  | - | - | - | - | X |
| *Stomatepia mongo* | CAM01_185 |  |  | - | - | - | - | X |
| *Stomatepia mariae* | CAM01_147 | ZSM 29835-36 | Lake Barombi Mbo, Cameroon | AJ844922 | AJ845096 | AJ845142 | X | - |
| *Stomatepia mariae* | CAM01_269 |  |  | AJ844923 | - | - | - | X |
| *Stomatepia mariae* | CAM01_146 |  |  | AJ844924 | AJ845097 | AJ845143 | X | X |
| *Stomatepia mariae* | CAM01_145 |  |  | AJ844925 | - | - | - | - |
| *Stomatepia mariae* | CAM01_214 |  |  | - | - | - | - | X |
| *Stomatepia mariae* | CAM01_144 | ZSM 29839-40, 29862 | Lake Barombi Mbo, Cameroon | AJ844926 | - | - | - | X |
| *Stomatepia pindu* | CAM01_138 |  |  | AJ844927 | - | - | - | - |
| *Stomatepia pindu* | CAM01_136 |  |  | AJ844933 | - | - | - | X |
| *Stomatepia pindu* | CAM01_135 |  |  | AJ844928 | AJ845098 | AJ845144 | X | X |
| *Stomatepia pindu* | CAM01_134 |  |  | AJ844930 | - | - | - | X |
| *Stomatepia pindu* | CAM01_137 |  |  | - | - | - | - | X |
| *Stomatepia pindu* | CAM01_263 |  |  | AJ844931 | AJ845099 | AJ845145 | X | X |
| *Stomatepia pindu* | CAM01_264 | ZSM 29851-53 | Lake Barombi Mbo, Cameroon | AJ844932 | - | - | - | X |
| *Pungu maclareni* | CAM01_120 |  |  | AJ844934 | AJ845100 | AJ845146 | X | X |
| *Pungu maclareni* | CAM01_123 |  |  | AJ844935 | AJ845101 | AJ845147 | X | X |
| *Pungu maclareni* | CAM01_128 |  |  | AJ844938 | - | - | - | X |
| *Pungu maclareni* | CAM01_124 |  |  | AJ844936 | - | - | - | - |
| *Pungu maclareni* | CAM01_126 |  |  | AJ844929 | - | - | - | - |
| *Pungu maclareni* | CAM01_127 |  |  | AJ844937 | - | - | - | X |
| *Pungu maclareni* | CAM01_129 | ZSM 29832-534, 29850, 29856 | Lake Barombi Mbo, Cameroon | AJ844939 | - | - | - | X |
| *Konia eisentrauti* | CAM01_297 |  |  | AJ844940 | AJ845102 | AJ845148 | X | X |
| *Konia eisentrauti* | CAM01_295 |  |  | AJ844941 | AJ845103 | AJ845149 | X | X |
| *Konia eisentrauti* | CAM01_216 |  |  | AJ844942 | - | - | - | X |
| *Konia eisentrauti* | CAM01_217 |  |  | AJ844943 | - | - | - | X |
| *Konia eisentrauti* | CAM01_219 |  |  | - | - | - | - | X |
| *Konia eisentrauti* | CAM01_218 |  |  | AJ844944 | - | - | - | X |
| *Konia dikume* | CAM01_048 | ZSM 29841-43, 29849, 29855 | Lake Barombi Mbo, Cameroon | AJ844945 | - | - | - | X |
| *Konia dikume* | CAM01_051 |  |  | AJ844946 | AJ845104 | AJ845150 | X | X |
| *Konia dikume* | CAM01_052 |  |  | AJ844947 | - | - | - | - |
| *Konia dikume* | CAM01_053 |  |  | AJ844948 | AJ845105 | AJ845151 | X | X |
| *Konia dikume* | CAM01_046 |  |  | - | - | - | - | X |
| *Konia dikume* | CAM01_057 |  |  | AJ844949 | - | - | - | X |
| *Myaka myaka* | CAM01_075 | ZSM 29844-47, 29861 | Lake Barombi Mbo, Cameroon | AJ844950 | AJ845106 | AJ845152 | X | X |
| *Myaka myaka* | CAM01_076 |  |  | AJ844951 | AJ845107 | AJ845153 | X | X |
| *Myaka myaka* | CAM01_082 |  |  | AJ844953 | - | - | - | X |
| *Myaka myaka* | CAM01_084 |  |  | AJ844954 | - | - | - | X |
| *Myaka myaka* | CAM01_078 |  |  | AJ844952 | - | - | - | - |
| *Myaka myaka* | CAM01_083 |  |  | - | - | - | - | X |
| *Sarotherodon lohbergeri* | CAM01_351 | ZSM 29821-25 | Lake Barombi Mbo, Cameroon | AJ844955 | - | - | - | X |
| *Sarotherodon lohbergeri* | CAM01_350 |  |  | AJ844956 | - | - | - | X |
| *Sarotherodon lohbergeri* | CAM01_226 |  |  | AJ844958 | AJ845109 | AJ845155 | X | X |
| *Sarotherodon lohbergeri* | CAM01_349 |  |  | AJ844959 | - | - | - | X |
| *Sarotherodon lohbergeri* | CAM01_224 |  |  | AJ844957 | AJ845108 | AJ845154 | X | X |
| *Sarotherodon lohbergeri* | CAM01_227 |  |  | - | - | - | - | X |
| *Sarotherodon lohbergeri* | CAM01_354 |  |  | AJ844960 | - | - | - | X |
| *Sarotherodon steinbachi* | CAM01_239 | ZSM 29828-31 | Lake Barombi Mbo, Cameroon | AJ844961 | AJ845110 | AJ845156 | X | X |
| *Sarotherodon steinbachi* | CAM01_241 |  |  | AJ844962 | - | - | - | X |
| *Sarotherodon steinbachi* | CAM01_235 |  |  | AJ844965 | AJ845111 | AJ845157 | X | X |
| *Sarotherodon steinbachi* | CAM01_244 |  |  | AJ844967 | - | - | - | - |
| *Sarotherodon steinbachi* | CAM01_231 |  |  | AJ844963 | - | - | - | X |
| *Sarotherodon steinbachi* | CAM01_233 |  |  | AJ844964 | - | - | - | X |
| *Sarotherodon steinbachi* | CAM01_243 |  |  | AJ844966 | - | - | - | X |
| *Sarotherodon caroli* | CAM01_153 | ZSM 29808-10, 29814, 29817-18, 29820, 29827, 29857, 29859 | Lake Barombi Mbo, Cameroon | AJ844968 | - | - | - | - |
| *Sarotherodon caroli* | CAM01_154 |  |  | AJ844969 | - | - | - | X |
| *Sarotherodon caroli* | CAM01_165 |  |  | AJ844974 | - | - | - | - |
| *Sarotherodon caroli* | CAM01_155 |  |  | AJ844970 | - | - | - | X |
| *Sarotherodon caroli* | CAM01_158 |  |  | AJ844971 | AJ845112 | AJ845158 | X | X |
| *Sarotherodon caroli* | CAM01_159 |  |  | AJ844972 | AJ845113 | AJ845159 | X | X |
| *Sarotherodon caroli* | CAM01_160 |  |  | AJ844973 | - | - | - | X |
| *Sarotherodon linnellii* | CAM01_273 | ZSM 29811-13, 29815-16, 29854 | Lake Barombi Mbo, Cameroon | AJ844978 | AJ845114 | AJ845160 | X | X |
| *Sarotherodon linnellii* | CAM01_262 |  |  | AJ844975 | - | - | - | X |
| *Sarotherodon linnellii* | CAM01_275 |  |  | AJ844980 | - | - | - | X |
| *Sarotherodon linnellii* | CAM01_272 |  |  | AJ844977 | - | - | - | - |
| *Sarotherodon linnellii* | CAM01_274 |  |  | AJ844979 | AJ845115 | AJ845161 | X | X |
| *Sarotherodon linnellii* | CAM01_277 |  |  | AJ844981 | - | - | - | - |
| *Sarotherodon linnellii* | CAM01_117 |  |  | AJ844976 | - | - | - | X |
| *Oreochromis niloticus* | CICH_007 | ZSM uncat | Genbank (mtDNA) and Ethiopia | AB018989 | AF317237 | AJ237397 | X | - |
| *Sarotherodon melanotheron „Import“* | SARO_008 | no voucher | Com. import prob. from Nigeria | - | - | - | X | - |
| *Sarotherodon melanotheron nigripinnis* | CAM01_443 | ZSM 29860 | Sanaga River at Edea, Cameroon | AJ844917 | AJ845084 | AJ845130 | X | - |
| *Sarotherodon galilaeus sanagaensis* | SARO_001 | ZSM 29858 | Sanaga River at Edea, Cameroon | AJ844918 | AJ845085 | AJ845131 | X | X |
| *Sarotherodon galilaeus multifasciatus* | SARO_ 002 | ZSM uncat. | Lake Bosumtwi, Ghana | AJ844984 | AJ845087 | AJ845133 | X | X |
| *Sarotherodon galilaeus multifasciatus* | CAM01_447 |  |  | AJ844982 | AJ845086 | AJ845132 | X | X |
| *Sarotherodon galilaeus multifasciatus* | CAM01_446 |  |  | AJ844983 | - | - | - | - |
| *Sarotherodon galilaeus „Meme“* | CAM00_002 | ZSM uncat. | Mémé River, Cameroon | AJ844985 | AJ845088 | AJ845134 | X | X |
| *Sarotherodon galilaeus „Meme“* | CAM00_007 |  |  | AJ844986 | AJ845089 | AJ845135 | X | X |
| *Sarotherodon galilaeus „Meme“* | CAM00_013 |  |  | AJ844987 | - | - | - | X |
| *Sarotherodon galilaeus „Meme“* | CAM00_015 |  |  | AJ844988 | - | - | - | X |
| *Sarotherodon galilaeus „Cross“* | CAM00_035 | ZSM uncat. | Cross River at Mamfé, Cameroon | AJ844989 | - | - | - | X |
| *Sarotherodon galilaeus „Cross“* | CAM00_032 |  |  | AJ844990 | AJ845090 | AJ845136 | X | X |
| *Sarotherodon galilaeus „Cross“* | CAM00_034 |  |  | AJ844991 | - | - | - | - |
| *Sarotherodon galilaeus „Cross“* | CAM00_036 |  |  | AJ844992 | - | - | - | X |
| *Sarotherodon galilaeus „Volta Noire“* | CAM01_452 | ZSM uncat | Black Volta River, Burkina Faso | AJ845006 | - | - | - | X |
| *Sarotherodon galilaeus „Volta“* | CAM01_450 | ZSM uncat | Volta River, Ghana | AJ845007 | - | - | - | X |
| *Sarotherodon galilaeus „Niger“* | CAM01_448 | ZSM uncat | Niger River at Bamako, Mali | AJ845009 | - | - | - | - |
| *Sarotherodon galilaeus „Niger“* | CAM01_449 |  |  | AJ845008 | AJ845093 | AJ845139 | X | X |
| *Sarotherodon sp. „mudfeeder“* | CAM00_082 | ZSM 29924-38, 30464 | Lake Ejagham, Cameroon | AJ844999 | - | - | - | X |
| *Sarotherodon sp. „mudfeeder“* | CAM00_083 |  |  | AJ845000 | AJ845092 | AJ845138 | X | X |
| *Sarotherodon sp. „mudfeeder“* | CAM00_084 |  |  | AJ845001 | - | - | - | X |
| *Sarotherodon sp. „mudfeeder“* | CAM00_085 |  |  | AJ845002 | - | - | - | X |
| *Sarotherodon sp. „mudfeeder“* | CAM00_088 |  |  | AJ845003 | - | - | - | - |
| *Sarotherodon sp. „mudfeeder“* | CAM00_089 |  |  | AJ845004 | - | - | - | - |
| *Sarotherodon sp. „mudfeeder“* | CAM00_087 |  |  | AJ845005 | - | - | - | - |
| *Sarotherodon sp. „bighead“* | CAM00_071 | ZSM 29929-34 | Lake Ejagham, Cameroon | AJ844993 | - | - | - | X |
| *Sarotherodon sp. „bighead“* | CAM00_072 |  |  | AJ844994 | AJ845090 | AJ845137 | X | X |
| *Sarotherodon sp. „bighead“* | CAM00_075 |  |  | AJ844995 | - | - | - | - |
| *Sarotherodon sp. „bighead“* | CAM00_076 |  |  | AJ844996 | - | - | - | - |
| *Sarotherodon sp. „bighead“* | CAM00_077 |  |  | AJ844997 | - | - | - | - |
| *Sarotherodon sp. „bighead“* | CAM00_073 |  |  | AJ844998 | - | - | - | X |
